# Animal-Free Chemical Safety Assessment

**DOI:** 10.3389/fphar.2016.00218

**Published:** 2016-07-21

**Authors:** George D. Loizou

**Affiliations:** Health Risks, Health and Safety Laboratory, Health and Safety ExecutiveBuxton, UK

**Keywords:** animal-free, safety, assessment, metabolomics, personalized, medicine, biosensors

## Abstract

The exponential growth of the Internet of Things and the global popularity and remarkable decline in cost of the mobile phone is driving the digital transformation of medical practice. The rapidly maturing digital, non-medical world of mobile (wireless) devices, cloud computing and social networking is coalescing with the emerging digital medical world of omics data, biosensors and advanced imaging which offers the increasingly realistic prospect of personalized medicine. Described as a potential “seismic” shift from the current “healthcare” model to a “wellness” paradigm that is predictive, preventative, personalized and participatory, this change is based on the development of increasingly sophisticated biosensors which can track and measure key biochemical variables in people. Additional key drivers in this shift are metabolomic and proteomic signatures, which are increasingly being reported as pre-symptomatic, diagnostic and prognostic of toxicity and disease. These advancements also have profound implications for toxicological evaluation and safety assessment of pharmaceuticals and environmental chemicals. An approach based primarily on human *in vivo* and high-throughput *in vitro* human cell-line data is a distinct possibility. This would transform current chemical safety assessment practice which operates in a human “data poor” to a human “data rich” environment. This could also lead to a seismic shift from the current animal-based to an animal-free chemical safety assessment paradigm.

## Introduction

In a paper titled “The feasibility of replacing animal testing for assessing consumer safety: a suggested future direction" Fentem et al. (2004) discussed how the new “omics” technologies; genomics, transcriptomics, proteomics and metabonomics could be used in the future to replace animal-based data in human chemical safety assessment. At that time, a major impediment to progress was that much of these data were generated in a clinical setting and not in abundance in the public domain. They recommended that making these data and information generally accessible in an ethical and legal way, could lead to the translation of experimental non-animal data that could be used in safety assessment (Fentem et al., 2004).

Much has changed in the research landscape since then. The expansions of the internet, allowing greater connectivity of devices and sensors, computational speed, cloud computing and multi-disciplinary collaborations, are the main developments that characterize these changes. For example, in recent years the speed of supercomputers has increased by several orders of magnitude, boasting processing speeds of 10^15^ floating-point operations per second which will soon reach 10^18^ floating-point operations per second (Witze, 2014). Without such computational power the production of approximately 1.8 zettabytes (10^21^) of genomic, epigenomic, transcriptomic, proteomic and metabolomic data generated each year, roughly doubling the world’s information resource every two years, would not be possible (Dearry, 2013). Indeed, more than 50,000 omics papers are published each year (Cote et al., 2014). Movements such as, Open Access in publishing (Bains, 2009), Open Source Initiative in software development^1^, Open Source engineered human tissue models (De Wever et al., 2015), the Open Phacts Foundation (Williams et al., 2012) and the need for multi-disciplinary approaches to the access, integration and analysis of big data sets (Schumacher et al., 2014; Alyass et al., 2015) has led to a burgeoning of collaborative research ^2^. This in turn has led to the proliferation of publicly available databases that include omics data for human disease, as well as survey and clinical assay data on human exposure and health outcomes (Zhu et al., 2008; Sakurai et al., 2011; Kim et al., 2012; Kotera et al., 2012; Kamburov et al., 2013; Wachter and Beissbarth, 2015). This new environment is leading to significant paradigm shifts in medicine and toxicology. Indeed, medicine is being transformed into a data science (Topol, 2010; Hood et al., 2015; Topol et al., 2015).

These changes could lead to the transformation of human chemical safety assessment from a “human data poor” to a “human data rich” arena with the consequent elimination of animal-based toxicology studies that currently underpin chemical safety assessment. In this review the components that could bring about an animal-free chemical safety assessment paradigm are discussed.

## Systems Biology

**FIGURE 1:**
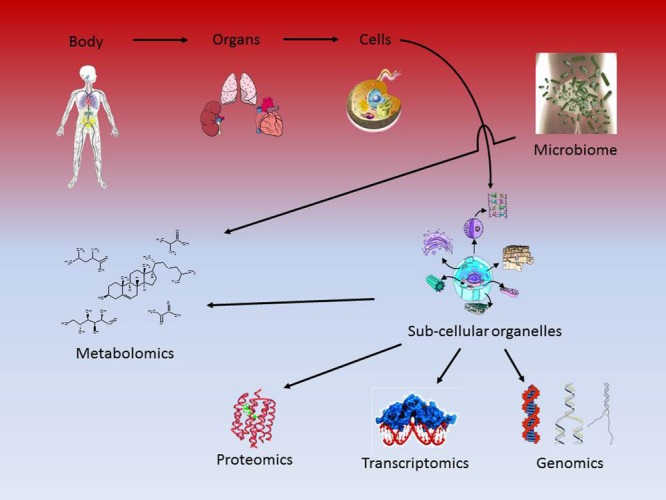
**The reductionist approach to the study of biology**.

Contemporary methods for the diagnosis of human disease originated in the late 19^th^ century, and are based on simple observational correlations between clinical syndromes and pathological analysis (Loscalzo et al., 2007; Loscalzo and Barabasi, 2011). Over the same period, research followed the reductionist approach that attempts to explain complex phenomena by defining the functional properties of the individual components that make up a system (Sobradillo et al., 2011). Consequently, the research focus progressed from the whole organism (anatomy) to the organs (physiology), cells (cell biology) and ultimately to subcellular molecular interactions (genes, proteins, lipids and metabolites; molecular biology) (**Figure 1**). This reductionist strategy is based on the assumption that many of the functions of the whole organism can be understood by knowing the properties of the component parts (Sobradillo et al., 2011). Both the approach to the diagnosis of disease and the reductionist strategy to research have made major contributions to our understanding of health and disease, however, they have inherent significant limitations. Current methods for diagnosing disease lack sensitivity for identifying preclinical disease (i.e., identifying precursor events that support early detection and treatment), and specificity in unequivocally defining disease (Loscalzo et al., 2007). The reductionist approach does not account for phenomena that emerge from the interactions of parts, and that appear as ‘coordinated’ functions of the individual components at higher levels of system organization (Sobradillo et al., 2011).

An alternative to the reductionist mind-set is the ‘systems biology’ perspective that integrates events at various levels of ‘system’ organization, and accounts for interactions of individual components and emerging properties that cannot be deduced from information on the single elements alone (Sobradillo et al., 2011).

Systems biology and systems medicine are characterized by the application of computational and mathematical modeling techniques that aim to unravel and understand the complexity of normal and diseased biological systems (Galas and Hood, 2009; Loscalzo and Barabasi, 2011; Chen and Snyder, 2012; Jack et al., 2013). They are biology-based, inter-disciplinary studies that deploy engineering approaches to discover emergent properties of cells, tissues and organs functioning as a system from the interactions between genetic, metabolic and cell signaling responses (Galas and Hood, 2009; Loscalzo and Barabasi, 2011; Chen and Snyder, 2012; Jack et al., 2013).

In systems biology, the implications of altered molecular and cellular components that result from exposure to chemical and non-chemical stressors, are studied and *integrated* across multiple levels of biological organization. That is, from genes to gene expression products, to alterations in biochemical pathways and networks and the propagation of effects from cells to tissues to organs and the whole body (Andersen et al., 2005; Zhang et al., 2010). Disease arises as a consequence of disease-perturbed networks in the diseased organ that propagate from one or a few disease-perturbed networks to many as the disease progresses. These initial disease perturbations may be due to genetic changes (e.g., mutations) and/or from exposure to stressors in the environment (e.g., infectious organisms, or chemicals). These perturbations alter the information expressed in these networks dynamically – and these altered dynamics of information flow explain the pathophysiology of the disease and suggest new approaches to diagnosis and therapy (Hood et al., 2012). By treating disease as a consequence of genetic and/or environmental perturbations of biological networks the systems approach also considers social and environmental influences that may impact health. The cross-talk of all networks is integrated in order to understand their functioning in the context of the individual (Hood and Friend, 2011; Hood et al., 2012, 2013, 2015; Smarr, 2012). Importantly, there is a growing body of evidence that these perturbations conform to biological patterns or ‘signatures’ that are associated with specific diseases (Nicholson and Holmes, 2006; Holmes and Nicholson, 2007; Holmes et al., 2008; Nicholson et al., 2008; Bouhifd et al., 2013).

## The Internet of Things, the Mobile Phone and Personalized Medicine

“Medicine is undergoing a revolution that will transform the practice of healthcare in virtually every way” (Hood et al., 2013). The systems approach to disease is beginning to change healthcare by deploying technologies that permit the rapid sequencing of an individual human genome and the quantification of “units of biological information” such as single genes, single molecules, single cells and single organs to provide disease relevant information on health or disease for the individual. This is resulting in an explosion of patient data that is transforming “traditional biology and medicine” into an information science (Hood and Friend, 2011; Hood et al., 2012, 2013, 2015; Smarr, 2012). By harnessing the capabilities of computational analysis of “big data” the digital revolution is transforming healthcare just as it has already transformed communications, finance, retail and information technology (Hood and Friend, 2011; Hood et al., 2012, 2013, 2015). The digital revolution is making the management and analysis of extremely large biological and environmental datasets tractable and it is driving the invention of personal monitoring devices that can digitize biological information, thus enabling, the individual assessment of wellness and disease commonly described as personalized medicine (Hood and Friend, 2011; Hood et al., 2012, 2013, 2015; Smarr, 2012).

Personalized Medicine, Stratified Medicine, Precision Medicine^3^^,^^4^ and P4 Medicine are interchangeable terms for systems medicine approaches to individualized healthcare (Topol, 2010; Hood et al., 2012, 2013; Smarr, 2012; Collins and Varmus, 2015; Topol et al., 2015). Personalized Medicine is a medical model that separates patients into different groups - with medical decisions, practices, interventions and products being tailored to the individual patient based on their predicted response or risk of disease. It is emerging from the convergence of systems medicine, the healthcare-focussed derivative of systems biology and the digital revolution (Hood et al., 2013). It’s proponents ascribe this revolution to the digital transformation of medical practice as being due to the “coalescence of the rapidly maturing digital, non-medical world of mobile (wireless) devices, cloud computing and social networking with the emerging digital medical world of genomics, biosensors and advancing imaging” (Topol, 2012). Described as the “greatest convergence in our history,” this revolution has become possible because of the exponential growth of the Internet of Things (IoT) and the global popularity and remarkable decline in cost of the mobile phone^5^ (Topol, 2010, 2012; Mak, 2015).

The IoT has been defined as a “global infrastructure for the information society, enabling advanced services by interconnecting (physical and virtual) things based on existing and evolving interoperable information and communication technologies”^6^. Fundamentally, the IoT comprises sensors, which are increasingly being embedded into smartphones and wearable devices interconnected via the internet. The ability to pack 19 million transistors into integrated circuits that occupy a 16 nm space explains how there are more than 2 billion transistors in some current smartphones (Topol et al., 2015). Current devices are already able to “digitize the biology of a human being” with the use of wearable sensors to quantify physiological metrics such as vital signs or relevant features of an individual’s environment, provide high definition images of the anatomy, and elucidate an individual’s biology by sequencing their DNA, RNA, microbiome and epigenome (Topol, 2014). In the next 5 years, individuals with hypertension and diabetes could have their blood pressure and glucose levels continuously monitored, most routine laboratory tests may be obtainable with smartphone kits and time series measurements of key biochemical variables should be feasible (Smarr, 2012; Topol et al., 2015). Billions of data points from each individual will be uploaded to a virtual cloud where sophisticated algorithms will decipher ‘signal’ from noise generated by the complexities of health and disease (Hood and Friend, 2011; Hood et al., 2012, 2013, 2015; Smarr, 2012). Unsurprisingly, this would constitute a seismic shift from the current “healthcare” model to a “wellness” paradigm that is predictive, preventative, personalized and participatory (Hood and Friend, 2011; Hood et al., 2012, 2013, 2015; Smarr, 2012).

Central to personalized medicine is biomarker tracking, specifically, the monitoring of time series measurements of key biochemical variables in an individual. Soon it may be possible to use integrated microfluidic chip technology to rapidly measure a panel of plasma proteins from a finger prick volume of whole blood. This could provide inexpensive, point-of-care, informative clinical diagnosis (Fan et al., 2008; Hood and Friend, 2011; Smarr, 2012; Hood et al., 2013, 2015). This technology could lead to the identification of organ-specific blood protein “fingerprints” that distinguish normal functioning from disease-perturbed biological networks (Hood et al., 2012, 2013). Such fingerprints or “signatures” are not confined to proteins. In fact, the field of metabolomics has tremendous potential for the identification of pre-symptomatic, diagnostic and prognostic metabolic signatures of disease, toxicity and exposure to environmental pollutants (Nicholson and Holmes, 2006; Holmes and Nicholson, 2007; Holmes et al., 2008; Nicholson et al., 2008; Bouhifd et al., 2013).

The wellness paradigm may be characterized by the longitudinal monitoring of integrative personal omics profiles (iPOP) which combine genomics, transcriptomics, proteomics, metabolomics and autoantibody profiles (Stanberry et al., 2013). In this approach, changes in metabolite expression levels reflect differential expression of biological pathways and are associated with disease (Stanberry et al., 2013; Guo et al., 2015).

## Metabolomics

Genetics alone cannot fully explain differences in disease predisposition, (Nicholson, 2006). Only about 5–10% of total human genetic variance occurs across populations and ethnic groups, although disease distributions and drug toxicity may vary greatly. Broadly speaking, genomics does not account for differences in phenotype (Holmes et al., 2008). Although a gene may be expressed and a protein may be synthesized, this protein may not be in the proper form to induce a metabolic change and therefore induce a phenotypic effect. The epigenome, which consists of non-sequence-based modifications, such as DNA methylation, is heritable and may affect normal phenotypes and predisposition to disease (Feinberg, 2010; Feinberg et al., 2010). Indeed, epigenetic changes have been shown to have a strong relationship with cancer and other common diseases (Feinberg et al., 2010).

Critical illness is characteristically the loss of metabolic homeostasis (Serkova et al., 2011; Mastrangelo et al., 2014). Monitoring the fluctuations of endogenous, low-molecular weight molecules in blood (plasma and serum) and urine is an important way to detect various human pathologies such as, cancer, cardiovascular disease, diabetes and drug and chemical toxicity (Serkova et al., 2011). Thus, metabolomics, or metabolic profiling, is the study of the quantitative description of all low-molecular-weight (<1 kDa) components in a biological sample. These may consist of metabolites solely under endogenous control and may also involve those originating from exogenous sources (microbiome, diet, drugs, and environmental pollutants).

The combination of genes and environment contribute to the effects observed in the metabolome as do factors such as gender, age, diet, exposure to xenobiotics and products of the gut microbiota (Mastrangelo et al., 2014). In addition, metabolomics involves the quantification of metabolites to track the developing response to a stimulus, has the advantage of being high-throughput and currently provides the best approach to delineating and understanding a biological mechanism preceding an effect (Kosmides et al., 2013; Mastrangelo et al., 2014).

**FIGURE 2:**
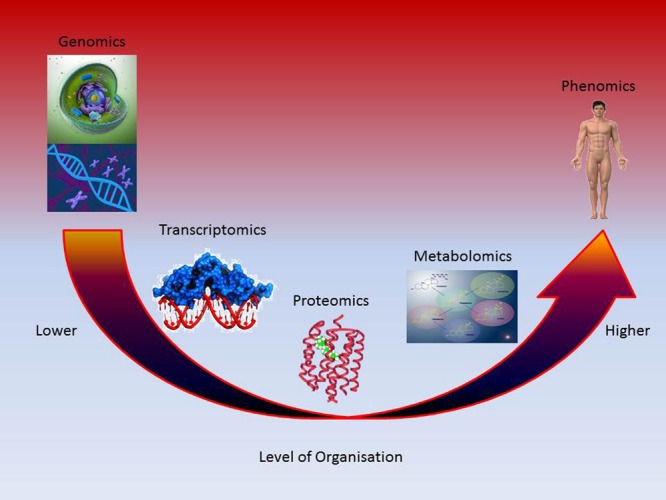
**Metabolic profiling reflects the collective effects of lower levels of organization**.

The advantage of metabolomics is that it allows the evaluation of changes at a higher level of organization, that is, closer to the phenotype which therefore, should provide a more reliable indication on the state of health of the individual (**Figure 2**). This is possible because endogenous small molecules are at the top of the systems biology continuum and reflect and magnify (several thousands of times) perturbations that occur at the genomic, transcriptomic and proteomic level (Raamsdonk et al., 2001; Wishart, 2012). Indeed, metabolomics data are needed to construct powerful top–down systems biology tools that link the omics disciplines (Coen et al., 2008). In this respect, the ability to link the information provided by the different omics data and build a pathway of toxicity (PoT) linking an external stressor induced perturbation to a disease endpoint is analogous to the development of chemically agnostic adverse outcome pathways (AOPs) proposed for use in chemical safety assessment (Ankley et al., 2010; Bouhifd et al., 2013; Burden et al., 2015; Athersuch, 2016; Edwards et al., 2016). It could provide novel information on phenotypic characteristics and therefore the potential to investigate the output of complex, interconnected networks (Kosmides et al., 2013).

Metabolomics is very sensitive, currently capable of detecting femtomolar to attomolar (10^-15^ to 10^-18^) changes in metabolite concentrations (Veenstra, 2012). Small dietary changes, increased physical activity, elevated stress or even variations in seasons can significantly alter metabolic profiles (Monte et al., 2014). Another advantage of metabolomics is that experimental and analytical variation in commonly used methods of metabolite measurement are several orders of magnitudes smaller than biological variation which confers robustness to metabolomic signatures (Keun et al., 2002; Maher et al., 2007).

## Targeted and Non-Targeted Metabolomics

There are two main strategies used in metabolomic studies: targeted or untargeted (Fiehn, 2001; Mastrangelo et al., 2014; Guo et al., 2015). Targeted approaches are generally used in the identification of potential direct or surrogate biomarkers of health, disease and mechanistic pathways and non-targeted approaches for the detection of broad classes of biochemical to provide a comprehensive functional phenotype integrating clinical phenotypes with genetic and non-genetic factors (Jones et al., 2012; Guo et al., 2015). Non-targeted studies require the application of bioinformatics and computational tools to analyze and interpret large and complex data (Jones et al., 2012).

There are three specific applications of metabolomics, non-targeted metabolic fingerprinting and metabolic profiling and targeted metabolic profiling (Kraly et al., 2009). Non-targeted metabolic fingerprinting seeks to measure a global profile of metabolites with identification of specific profiles based on pattern recognition. A major weakness of metabolic fingerprinting is the inability to identify specific biomarkers for a disease state or therapeutic endpoint. Metabolic profiling is the measurement of the full complement of low-molecular-weight metabolites and their intermediates, such as amino acids, carbohydrates and lipids, that reflects the dynamic response to genetic modification and physiological, pathophysiological and/or developmental stimuli (Clarke and Haselden, 2008). In targeted metabolic profiling one or two analytes are tracked with time and because of this is often excluded from the discussion of metabolomics. However, it is a very useful tool for understanding biological systems (Kraly et al., 2009).

## Metabolomics and the Microbiome

Metabolic profiling can also include the contribution from gut microorganisms, the microbiome. The microbiome comprises more than 100 trillion microorganisms belonging to 300–500 different species that live inside and on every human being (Guarner and Malagelada, 2003). The number of microorganisms in a healthy human adult are estimated to outnumber human cells by a ratio of ten to one^7^ and make up 1–3% of body mass^8^ (0.75–2.25 kg in a 75 kg person) and represents a confounding factor when interpreting genomic, proteomic or metabolomic response data (Nicholson et al., 2004; Nicholson, 2006; Kinross et al., 2008; Nicholson and Lindon, 2008). An Individual’s microbiome is unique and may share as little as 1% of the same type of bacteria with other people (Kinross et al., 2008) and may change with age, diet, drugs, disease and medical or surgical intervention (Kinross et al., 2008). The gut microbiome interacts with the other systems in the body and has metabolic, trophic and protective functions (Guarner and Malagelada, 2003; Kinross et al., 2009). It influences the levels of cytochrome P450 enzymes (Nicholson et al., 2004), has a significant role in obesity (Turnbaugh et al., 2006; Kinross et al., 2008; Li et al., 2008; Calvani et al., 2010) sepsis (MacFie et al., 1999; Shimizu et al., 2006), inflammatory bowel disease, irritable bowel syndrome and colon cancer (Guarner and Malagelada, 2003). The gut microbiome contributes to interindividual variability in drug toxicities and may contribute to the bioactivation of carcinogens that would not have been metabolized by the human cells (Nicholson et al., 2005). Metabolites arising from the gut microbiome merge with endogenous chemicals thereby altering the metabolome without having influenced gene and protein expression.

## Metabolite Signatures

In toxicology, extensive efforts are underway to identify signatures of toxicity which are patterns of metabolite changes predictive of the manifestation of toxicity and disease. These patterns are more commonly known as metabolite signatures (Bouhifd et al., 2013, 2014, 2015). Similarly, in clinical applications the prediction of xenobiotic toxicity or drug effects in an individual based on a mathematical model of pre-intervention metabolite signatures is known as pharmacometabolomics (Clayton et al., 2006). The identification of signatures associated with toxicity, drug effects and disease endpoints in a range of media, including; serum, plasma, urine, mucosa, exhaled breath, saliva, hair, tissue and cultured cells shows steady growth and could provide human data that may be used in chemical safety assessment (Bouhifd et al., 2013; Zhang et al., 2013a,b; Armitage and Barbas, 2014; Sulek et al., 2014). With the application of powerful bioinformatics and statistics, metabolite signatures can be used to identify a PoT which connects the molecular initiating event (MIE) of a toxicant with an adverse outcome (Ankley et al., 2010; Bouhifd et al., 2013; Vinken, 2013; Athersuch, 2016). The development of an underpinning mechanistic toxicology in the form of a perturbed PoT is a key concept for the implementation of the much vaunted Toxicity Testing for the 21st Century (NRC, 2007).

The following are just a few examples illustrating how the identification of *in vivo* metabolic signatures in pharmaceutical applications, environmental and occupational toxicology and in *in vitro* systems is a rapidly growing area that could provide actionable data for human chemical safety assessment.

In clinical applications metabolite signatures have been identified in a range of biological fluids that; can distinguish between patients with various cancers including; colorectal, pancreatic, gastric, liver, breast, ovarian, kidney, bladder, prostate, oesophageal, lung and oral and healthy controls (Armitage and Barbas, 2014), are consistent with early indications of diabetes, liver dysfunction and disruption of gut microbiome homeostasis in healthy volunteers (Guo et al., 2015), can distinguish between race and genotype in response to the antihypertensive drug atenolol (Wikoff et al., 2013), and were able to discriminate hepatitis B virus (HBV) infected subjects from healthy controls (Zhang et al., 2013a).

In environmental toxicology, metabolic profiling of urinary metabolites has been shown; to detect early effects of environmental and lifestyle exposure to cadmium in a human population (Ellis et al., 2012), to distinguish controls and alcohol consumers, but not smokers exposed to a complex mixture such as welding fumes (Kuo et al., 2012), to identify intermediate biomarkers of response to environmental/occupational concentrations of lead, cadmium and arsenic, in smelter workers (Dudka et al., 2014), to indicate oxidative stress-related effects in humans exposed to environmental concentrations of polycyclic aromatic hydrocarbons (PAHs) (Wang et al., 2015), and associate male infertility with arsenic exposure caused by a PoT involving oxidative stress and sexual hormone disruption (Shen et al., 2013).

Distinguishable metabolic signatures have been observed in *in vitro* cultured human fibroblast cells infected by herpes simplex virus type-1 (HSV-1) and human cytomegalovirus (HCMV) (Rabinowitz et al., 2011). *In vitro* metabolic signatures leading to hepatotoxicity in HepG2/C3a cells in microfluidic culture conditions which appeared consistent with literature reports of *in vivo* hepatotoxicity were identified (Choucha Snouber et al., 2013), and different *in vitro* hepatic metabolic signatures and pathways for ammonia, dimethylsulfoxide and paracetamol toxicity were identified in liver and kidney co-cultures (Shintu et al., 2012).

## Dose-Dependent Metabolite Signatures

To ensure public safety and environmental quality, regulatory agencies are required by law to undertake science-based safety and risk assessments of potential hazards (Burgoon and Zacharewski, 2008). These agencies use dose-response modeling to identify a Reference Point (RP), also known as a point-of-departure (PoD), which is the point of transition on the dose-effect curve, to derive a health-based guidance value (Sand et al., 2006, 2012; Burgoon and Zacharewski, 2008). Therefore, the identification of dose-dependent changes in metabolite signatures would permit the use of such data in the current safety assessment paradigm (European Food Safety Authority, 2014). Encouraging developments in dose-dependent changes in metabolic biomarkers in both *in vivo* and *in vitro* studies are increasingly reported. For example, dose-dependent changes were observed in urinary metabolite biomarkers of male infertility in Han Chinese men following environmental exposure to arsenic, and in cadmium-induced renal toxicity in Chinese women (Gao et al., 2014). In addition, the metabolomic changes observed in the latter study were sufficiently distinct to allow the differentiation of cadmium-induced renal toxicity from subjects with chronic kidney disease (Gao et al., 2014). In male Sprague Dawley rats treated with 0.5 or 2 mg/kg HgCl_2_ [mercury(II) chloride] maximal and marked kidney tubule necrosis was observed by 48 h post exposure at the high dose and modest injury at the low dose (Griffin and Bollard, 2004). *In vitro*, organ-specific, dose-dependent, predictive, compound-specific metabolite signatures for ammonia and paracetamol toxicity were observed in microfluidic liver and kidney co-cultures (Shintu et al., 2012).

The use of metabolite signatures in the safety assessment process could be possible if signatures observed *in vivo* can be reproduced in appropriate *in vitro* systems in which dose-response relationships could be more easily observed and measured (European Food Safety Authority, 2014). There are encouraging developments in the area.

## Microfluidics and Biochips

Common laboratory practice is to use two-dimensional (2D) cell culture techniques, that is, to grow cells on a flat substrate such as a petri dish or microtiter plate (van Duinen et al., 2015). In three-dimensional (3D) cell culture techniques cells are permitted to grow or interact with their surroundings in all three dimensions and have been shown to be an improvement on 2D cultures (van Duinen et al., 2015). For example, apical-basal polarization (Schoenenberger et al., 1994), lumen formation (Debnath et al., 2003), reduced proliferation and increased differentiation (Weaver et al., 1997) and numerous changes in RNA and protein expression (Lin and Bissell, 1993).

Yet existing 2D and 3D cell culture models do not fully recapitulate subtle organ-specific variations in the *in vivo* microenvironment (Huh et al., 2012). *In situ*, cells experience organ-specific dynamic variations in spatiotemporal chemical gradients and mechanical forces (e.g., cyclic strain, compression, fluid shear stresses) in their local tissue microenvironment that are crucial governors of their survival, growth and function. Thus, many fundamental aspects of cell behavior are mechanosensitive, including adhesion, spreading, migration, gene expression and cell–cell interactions (Jansen et al., 2015). Integrin-mediated mechanosensing feeds into cell fate decisions by activating various downstream signaling cascades connected to gene expression (Jansen et al., 2015).

Microfluidic techniques are based on micrometer-sized, hollow channels lined with living cells arranged to recreate tissue- and organ-level physiology which are continuously perfused with nutrient medium (Huh et al., 2010, 2012, 2013; Tseng et al., 2014; van Duinen et al., 2015). These technologies, also known as biochips and are about the size of a computer memory stick, further increase the physiological relevance of 3D cell culture by enabling spatially controlled co-cultures e.g., liver and kidney, perfusion flow and spatial control over signaling gradients (Smith et al., 2013; Tseng et al., 2014; van Duinen et al., 2015). The detection of a metabolomic signature in a co-cultured biochip should, in theory, be similar to an *in vivo* blood or urine metabolomic signature as they are all aggregate responses to a chemical stressor.

When coupled to metabolomics and intracellular gene and protein levels, biochips have the potential to provide a functional cell response. They can behave as “biosensor” systems when combined with metabolomic studies of organ culture media that may be useful in a high-throughput small-molecule screening approach (Prot et al., 2012). Indeed, biochips are being developed for high-throughput assay development (Prot and Leclerc, 2012; Prot et al., 2012; Trietsch et al., 2013). In addition, advances in real-time quantification of changes in intracellular metabolic activities have the potential to vastly improve the prediction of current and future cellular phenotypes based on metabolomic signatures (Heinemann et al., 2014). A proof-of-principle microfluidic-based inline small molecule extraction system which allows for continuous metabolomics analysis of living systems has been developed. This technology could detect cyclic patterns and forecastable metabolic trajectories. Metabolic oscillations and predictable transitions in both growth and stress related changes in *E. coli* and ovine whole blood could be observed and measured (Heinemann et al., 2014). The combination of recent advances in stem cell biology, such as induced pluripotent stem cells (Moreno et al., 2015) and organoid technology (Astashkina et al., 2012), with microfluidic 3D cell culture will lead to the implementation of personalized medicine and companion diagnostics in the next 5 years (van Duinen et al., 2015).

However, specific metabolite signatures have been observed that are a cellular response to the culture mode and cellular environment in biochips (Choucha Snouber et al., 2013; Sturla et al., 2014). For example, a cytoprotective cell response was induced in HepG2/C3a cells by the microfluidic biochip conditions (Prot et al., 2011). Also, there are differences in response between biochips and conventional plate cultures (Prot et al., 2012). The latter may occur due to differences in mechanosensing (Huh et al., 2012; Jansen et al., 2015). These must be distinguished from specific signatures that are consistent with those observed *in vivo* (Prot et al., 2012). Nonetheless, transcriptomic and proteomic signatures of acetaminophen toxicity observed in cultured HepG2/C3A cells in a microfluidic biochip study have been shown to be similar to those reported *in vivo* (Prot et al., 2011). Many more examples like that of acetaminophen, encompassing a representative chemical space, are required in order to provide the evidence base to replace animal-based toxicity testing.

## Validation of Biochip Data

Validation is the independent assessment of the scientific basis, the reproducibility, and the predictive capacity of a test. Currently, the validation of *in vitro* models is a significant challenge in drug candidate and toxicity screening. High percentages of new chemicals and biological entities still fail late-stage human drug testing, or receive regulatory “black box” warnings, or are removed from the market for safety reasons after regulatory approval (van Duinen et al., 2015). There are a number of reasons for the perception that *in vitro* cell-based assays and subsequent preclinical *in vivo* studies do not yet provide sufficient pharmacological and toxicity data or reliable predictive capacity for understanding drug candidate and environmental chemical performance *in vivo* (Adler et al., 2011; Astashkina et al., 2012; Piersma et al., 2014). The discussion of the regulatory acceptance of *in vitro* data for safety assessment is beyond the scope of this review. However, the reader may find the reviews of Adler et al. (2011) and Piersma et al. (2014) useful.

A key problem for the novel technologies is the absence of a point of reference, i.e., a “traditional test” or “gold standard.” In the absence of reference data, “scientific validation” needs to be stressed. This would involve a systematic review of the extent to which a given test reflects current scientific understanding. In the case of toxicity, this would require review of established modes of action (MOA), PoT and AOPs. This is in contrast to traditional validation, which largely considers the test system as a black box and compares the results obtained therein with those of another black box, the animal model(s) (Pamies et al., 2014). However, because of their origin human-derived 3D cell culture models are expected to be better predictors of clinical and toxic outcome than animal models (van Duinen et al., 2015). Retrospective validation based on clinical results for success or failure of compounds with regards to toxicology should be used as RPs for validation (van Duinen et al., 2015). Nonetheless, more human toxicity data and high-quality *in vivo* data are critical in assessing the true predictive power of *in vitro* data-based models of *in vivo* toxicity (Huang et al., 2016). Although historically these data, in many cases are not publicly available, particularly for drugs, there are increasingly numerous new freely available databases that may provide such data, e.g., the Human Metabolome Database (Wishart et al., 2013) and Consensus Path Database (ConsensusPathDB-human) (Kamburov et al., 2013). Therefore, an alternative validation strategy would be to compare biochemical changes between an *in vitro* model system and *in vivo* human interaction networks such as gene, protein, signaling, metabolic and drug-target interactions as well as gene regulation and biochemical pathway perturbations (Dumas, 2012; Kamburov et al., 2013; European Food Safety Authority, 2014).

## Characterisation of Human Exposure

The estimation of human exposure is of fundamental importance in the evaluation of the relevance and interpretation of dose-response data for toxicity in the assessment of health risks (Thomas et al., 2013). Therefore, a PoD determined in an *in vitro* system must be extrapolated to an *in vivo* PoD, which in turn must be related to an administered dose or tissue dose arising from human exposure (Thomas et al., 2013; Wetmore et al., 2015).

Human exposure may be estimated from the measurement of parent chemical concentrations in the blood or urinary metabolite concentrations from which exposure concentrations can be inferred using reverse dosimetry (Tan et al., 2006; Lyons et al., 2008; McNally et al., 2012, 2014). This approach could be appropriate in the case of blood parent chemical or urinary metabolite concentrations of known environmental pollutants measured as part of a metabolic profile.

For chemicals without biological monitoring data, as would be the case with a PoD derived from biochips, high-throughput human exposure models are being developed which combine environmental fate and transport models with indoor or consumer exposure models (Arnot et al., 2010, 2012; Wambaugh et al., 2013, 2014, 2015; Wetmore et al., 2015). Comparison of the administered dose or tissue dose with human exposure predictions could provide a margin of exposure (MOE) approach that can help the shift from a hazard based- toward a more risk-based- methodology (Thomas et al., 2013; Wetmore et al., 2015).

## Bioinformatics: Pathway and Network Analysis

Omics data must distinguish changes and pathways associated with impending pathology versus benign adaptive changes that are responsive to the chemical but are not associated with toxicity (Harrill and Rusyn, 2008). More and more powerful data analytics required to distinguish biological signals from noise are increasingly available (Braun, 2014). Once identified metabolomic signatures provide relevance beyond clinical biomarkers as they represent a powerful means of identifying mechanisms of toxicity and disease (Wikoff et al., 2013; Zhang et al., 2013a).

The rapid proliferation of metabolomics studies has led to difficulties in the identification of compounds, their physiological role or toxicity or disease-specific pathway (Collins, 2004). The Human Metabolome Database or HMDB is a resource designed to address these issues. It is an open access database with up-to-date referential information about metabolites, metabolic pathways, biomarkers and reference NMR, MS/MS (tandem mass spectrometry), and GC-MS (gas chromatography mass spectrometry) spectra for compound identification (Wishart, 2007; Wishart et al., 2009, 2013, 2016). At the time of writing the HMDB contains 41,993 metabolite entries, more than 5000 normal and abnormal metabolite concentrations and nearly 800 metabolic and disease-associated pathways and dozens of cancer biomarkers (Wishart et al., 2016). However, currently only a fraction of the known human metabolome is linked to pathways and secondary processes such as gut microbiome-generated effects and lipid metabolism (Wikoff et al., 2009, 2013). Even with the growing number of knowledge-based metabolic pathway databases that can be used to reveal the higher-order systemic operation of cells, organs and whole organisms (Stobbe et al., 2014; Zhukova and Sherman, 2014) more comprehensive tools and databases specifically designed for network and pathway analysis using metabolomics data are required (Xia and Wishart, 2010a,b; Kankainen et al., 2011; Wikoff et al., 2013). For example, perturbed metabolic pathways have been identified by mapping transcriptomic, proteomic and metabolomics data signatures using freely available resources such as the KEGG database and Ingenuity canonical pathways (Prot et al., 2012; Posma et al., 2014). However, few network mapping programs consider that the typical mammal has metabolic contributions from symbiotic gut microbiota and even parasitic organisms (Gill et al., 2006; Nicholson et al., 2012). MetaboNetworks, a freely available tool for the identification of complex metabolic reaction networks, combines metabolic reactions from different organisms and allows the delineation and combination of reaction networks from selected and combined organisms (Posma et al., 2014).

Another promising web-based tool is the ConsensusPathDB-human where human *in vivo* signatures may be identified by interrogating 32 freely accessible databases accessed via a single portal (Kamburov et al., 2013). The ConsensusPathDB-human integrates interaction networks in humans including binary and complex protein–protein, genetic, metabolic, signaling, gene regulatory and drug-target interactions, as well as biochemical pathways (Kamburov et al., 2013).

However, confidence in the quality and reliability of omics data must be improved. Specifically, significant improvements are necessary in the sensitivity, accuracy and reproducibility of these data (Leung et al., 2013). Biological variation and differences in “time of capture” of samples and inter-laboratory variation can lead to a lack of reproducibility (Leung et al., 2013).

There are efforts under way that combine high quality omics and phenome data in the same database that are already demonstrating an impressive level of sophistication and predictive capability. Currently there is restricted access to the data but they do demonstrate what is possible. For example, the Clinical Genome Resource, which was set up by the US National Human Genome Research Institute, is a database of disease-related variants, and contains information that could guide medical responses to these variants as well as the evidence supporting those associations (Rehm et al., 2015). Genomics England, which runs the 100,000 Genomes Project, aims to bolster progress in this area by establishing ‘clinical interpretation partnerships’: doctors and researchers will collaborate to establish robust models of diseases that can potentially be mapped to specific genetic alterations (Eisenstein, 2015). This will be achieved by building a database of clinical data with matching rich phenotype data from patient records^9^. Data will remain in a secure environment within which researchers will work (Siva, 2015)^10^^,^^11^. The 100,000 Genomes Project provides a monthly update of the number whole human genomes sequenced. As of first February 2016, 6,597 genomes have been sequenced^12^.

The Health Nucleus offered by Human Longevity Inc.^13^ uses whole genome sequence analysis, advanced clinical imaging and innovative machine learning – combined with a comprehensive curation of personal health history – to deliver the most complete picture of individual health. Currently, the database contains 20,000 genomes with matching phenomes with the view of expanding to over one million. The larger the database the more effective the correlations because the 6.5 billion bases in each individual’s DNA differs from another individual by just 3%. The phenome data is generated using the most advanced techniques to measure unique body structures and metabolomics profiles. Machine learning techniques are used to uncover associations. They claim a level of sophistication where genomes can be matched to photographs and voice recordings matched to sex, age, and height and face shape. In the “Face Project” they claim to have matched 100 photographs to 100 genomes ^14^^,^^15^.

As with all new developments a global initiative involving industry, regulatory agencies and academic institutions is required to standardize ‘omics’ methods and reach a consensus on the reliability and interpretation of endpoints (Leung et al., 2013).

## In Vitro to In Vivo Extrapolation

Whilst differentially expressed genes, proteins and metabolites provide a powerful means of understanding mechanisms (specific regulatory or signaling networks altered by treatments) that connect molecular and cellular changes at the tissue level, they are nonetheless “static” lists and therefore cannot provide a complete understanding of physiological processes or of toxicity. In order to replace *in vivo* animal testing and provide a quantitative, dynamic, mechanistic and predictive toxicology, concentration-response relationships observed and measured in biochips must be extrapolated to *in vivo*. This can be done with physiologically-based pharmacokinetic (PBPK) models (Loizou and Hogg, 2011; McNally et al., 2011, 2012; Prot and Leclerc, 2012; Coecke et al., 2013; Bessems et al., 2014; Sturla et al., 2014).

A PBPK model is an independent, structural model, comprising compartments that correspond directly and realistically to the organs and tissues of the body and connected by the cardiovascular system. They are mathematical descriptions of biological systems, in this case the human body, which are translated into computer code and solved computationally. They are frameworks that can capture our understanding of the science underlying the biological processes that lead to disease (McNally et al., 2011). The principle application of PBPK models is in the prediction of the appropriate form of the target tissue dose, or *dose-metric*, of the parent chemical or its reactive metabolite(s). The dose-metric must capture the critical biochemical steps that lead to the moiety causing the effect at the target site. Such mechanisms may take place within any compartment, e.g., blood, organ or sub-cellular compartment. Use of an appropriate dose-metric in chemical safety assessment calculations provides a better basis for relating the observed effects to the external or administered exposure concentration of the parent chemical (Conolly and Butterworth, 1995; Barton et al., 1998; IGHRC, 1999; Johanson et al., 1999; Andersen, 2003; Clewell and Clewell, 2008; Lipscomb and Poet, 2008).

Physiologically-based pharmacokinetic models can be used for forward or reverse dosimetry. The former converts inhalation, dermal exposure, oral or intravenous administration of a chemical to a target-tissue dose; the latter can reconstruct exposure or dose from parent chemical and/or metabolite(s) in urine, blood or *in vitro* surrogates of organ or tissue concentration (Tan et al., 2006; Clewell et al., 2008; Lyons et al., 2008; Louisse et al., 2012; McNally et al., 2012, 2014; Bessems et al., 2014). Therefore, PBPK models can be used to translate a RP derived from concentration-response relationships measured in biochips to a plausible distribution of human *in vivo* concentrations. This can be achieved by linking a PBPK model with Bayesian inference where replacing single point values for model parameters with informative prior distributions converts a deterministic model to a population-based model (McNally et al., 2012, 2014).

## Regulatory Acceptance

In the USA regulatory action must be defensible in court where, in the absence of the preferred proof that something, “is or is not true,” the supporting arguments are based on “precedent and expert opinion.” Regulators will change their actions when expert scientific opinion supports the use of alternative models over animal models, and regulatory action based on those alternative models can be defended in court i.e., regulators take their cues from expert scientists who provide them with legally defensible actions, not the other way around.

In the absence of a way to measure the “accuracy” of a new test versus existing animal test results, the default is to prove that an alternative-to-animals test produces results that are “similar” or “comparable” to the previous animal studies. If the results are different, then the alternative system cannot replace the animal studies, i.e., current practice and historical precedent win.

The emergence of human data, such as, chemical body burdens (i.e., full chemical and metabolite profiles) and biomarkers of effects for health status or steps along an AOP progression should change the current paradigm. The burden of proof to “validate” a new test should not require comparison with animal data, but should be which test provides the most accurate result to best protect public health. More accurate estimates of risk to protect public health based on human *in vivo* data must be considered more relevant and less uncertain than an estimate based on current practice which is derived from a few animal test results, primarily in rodents, adjusted by uncertainty factors which are scientifically poorly supported.

In Europe, the regulatory objective in not to obtain the most accurate estimate of risk, rather it is to drive the control of exposure to a level at which there is confidence of no significant risk. Regulatory acceptance is based more on understanding, transparency and robustness of new approaches and adherence with the stipulations of the regulations. European regulatory authorities must be confident in new technologies in order to adopt them and would generally do so without reference to court proceedings.

## The Near Foreseeable Future

If “foreseeable” refers to a range of time for which forecasts are possible and “forecasting” is to calculate or predict (some future event or condition) usually as a result of study and analysis of available pertinent data, then the next 5–10 years should see the transformation of occupational and environmental toxicology from a human data poor to a human data rich discipline. This transformation will come about through the coalescence of systems medicine and the digital revolution, the components of which, will in turn coalesce with the high-throughput *in vitro* systems- based toxicity testing paradigm proposed in the US National Research Council vision and strategy for future toxicity testing and safety assessment (NRC, 2007). The generation of human data that may be used in chemical safety assessment continues. For example, the development of a fully integrated wrist-band sensor for *in situ* analysis of sweat can provide real-time assessment of the physiological state of human subjects and may represent a platform for the development of a wide range of personalized diagnostic and physiological monitoring applications (Gao et al., 2016). Human sweat is a medium considered to be rich in physiological information (Sonner et al., 2015). It is reasonable to predict that the sophisticated sampling technology developed for such a device could be used to provide sweat samples for frequent, non-invasive metabolic profiling. Likewise, the development of relatively low-cost “electronic noses” for the non-invasive analysis of volatile organic compounds (VOCs) signatures in exhaled breath for the early detection of various cancers and other diseases must also bode well for the near future (Konvalina and Haick, 2013; Rattray et al., 2014; Krilaviciute et al., 2015; Gasparri et al., 2016).

Optimism for the development of an animal-free chemical safety assessment paradigm based on the identification of pre-symptomatic, diagnostic and prognostic metabolic signatures of toxicity and disease using non-invasive or minimally invasive biosensors appears to be justified.

## Author Contributions

This review is entirely the work of GL.

## Conflict of Interest Statement

The authors declare that the research was conducted in the absence of any commercial or financial relationships that could be construed as a potential conflict of interest.

## Footnotes

1 http://opensource.org/history (Accessed on 20 May 2016)

2 http://www.scienceeurope.org/uploads/PublicDocumentsAndSpeeches/SCsPublicDocs/Workshop_Report_MED_Big_Data_web.pdf (Accessed on 20 May 2016)

3 http://www.acmedsci.ac.uk/more/events/stratified-personalised-or-p4-medicine-a-new-direction/ (Accessed on 20 May 2016)

4 https://www.nih.gov/precision-medicine-initiative-cohort-program (Accessed on 20 May 2016)

5 http://www.economist.com/news/business/21600134-smartphones-reach-masses-host-vendors-are-eager-serve-them-rise-cheap (Accessed on 20 May 2016)

6 http://www.itu.int/ITU-T/recommendations/rec.aspx?rec=y.2060 (Accessed on 20 May 2016)

7 https://www.bcm.edu/departments/molecular-virology-and-microbiology/research/the-human-microbiome-project (Accessed on 20 May 2016)

8 http://www.nih.gov/news-events/news-releases/nih-human-microbiome-project-defines-normal-bacterial-makeup-body (Accessed on 20 May 2016)

9 http://www.genomicsengland.co.uk/the-100000-genomes-project/ (Accessed on 20 May 2016)

10 http://www.genomicsengland.co.uk/ (Accessed on 20 May 2016)

11 https://youtu.be/gW6CUCYw7Vs (Accessed on 20 May 2016)

12 http://www.genomicsengland.co.uk/the-100000-genomes-project-by-numbers/ (Accessed on 20 May 2016)

13 http://tinyurl.com/nehe3e4 (Accessed on 20 May 2016)

14 https://youtu.be/D_JwU UTD-hk (at 7 min 50 s) (Accessed on 20 May 2016)

15 https://youtu.be/4ywjThNbfYk (Accessed on 20 May 2016)

## References

[B1] Adler S. Basketter D. Creton S. Pelkonen O. van Benthem J. Zuang V. (2011). Alternative (non-animal) methods for cosmetics testing: current status and future prospects-2010. *Arch. Toxicol.* 85 367–485. 10.1007/s00204-011-0693-2 21533817

[B2] Alyass A. Turcotte M. Meyre D. (2015). From big data analysis to personalized medicine for all: challenges and opportunities. *BMC Med. Genomics* 8:33 10.1186/s12920-015-0108-y PMC4482045 26112054

[B3] Andersen M. E. (2003). Toxicokinetic modeling and its applications in chemical risk assessment. *Toxicol. Lett.* 138 9–27. 10.1016/S0378-4274(02)00375-2 12559690

[B4] Andersen M. E. Thomas R. S. Gaido K. W. Conolly R. B. (2005). Dose-response modeling in reproductive toxicology in the systems biology era. *Reprod. Toxicol.* 19 327–337. 10.1016/j.reprotox.2004.12.004 15686868

[B5] Ankley G. T. Bennett R. S. Erickson R. J. Hoff D. J. Hornung M. W. Johnson R. D. (2010). Adverse outcome pathways: a conceptual framework to support ecotoxicology research and risk assessment. *Environ. Toxicol. Chem.* 29 730–741. 10.1002/etc.34 20821501

[B6] Armitage E. G. Barbas C. (2014). Metabolomics in cancer biomarker discovery: current trends and future perspectives. *J. Pharm. Biomed. Anal.* 87 1–11. 10.1016/j.jpba.2013.08.041 24091079

[B7] Arnot J. A. Brown T. N. Wania F. Breivik K. McLachlan M. S. (2012). Prioritizing chemicals and data requirements for screening-level exposure and risk assessment. *Environ. Health Perspect.* 120 1565–1570. 10.1289/ehp.1205355 23008278 PMC3556628

[B8] Arnot J. A. Mackay D. Parkerton T. F. Zaleski R. T. Warren C. S. (2010). Multimedia modeling of human exposure to chemical substances: the roles of food web biomagnification and biotransformation. *Environ. Toxicol. Chem.* 29 45–55. 10.1002/etc.15 20821418

[B9] Astashkina A. Mann B. Grainger D. W. (2012). A critical evaluation of in vitro cell culture models for high-throughput drug screening and toxicity. *Pharmacol. Ther.* 134 82–106. 10.1016/j.pharmthera.2012.01.001 22252140

[B10] Athersuch T. (2016). Metabolome analyses in exposome studies: profiling methods for a vast chemical space. *Arch. Biochem. Biophys.* 589 177–186. 10.1016/j.abb.2015.10.007 26494045

[B11] Bains S. (2009). Open access movement. *Concept* 1.

[B12] Barton H. A. Andersen M. E. Clewell H. J. III (1998). Harmonisation: developing consistent guidelines for applying mode of action and dosimetry information to cancer and noncancer risk assessment. *Hum. Ecol. Risk Assess.* 4 74–115. 10.1080/10807039891284226

[B13] Bessems J. G. Loizou G. Krishnan K. Clewell H. J. III Bernasconi C. Bois F. (2014). PBTK modelling platforms and parameter estimation tools to enable animal-free risk assessment: recommendations from a joint EPAA - EURL ECVAM ADME workshop. *Regul. Toxicol. Pharmacol.* 68 119–139. 10.1016/j.yrtph.2013.11.008 24287156

[B14] Bouhifd M. Andersen M. E. Baghdikian C. Boekelheide K. Crofton K. M. Fornace A. J. (2015). The human toxome project. *Altex* 32 112–124. 10.14573/altex.1502091 25742299 PMC4778566

[B15] Bouhifd M. Hartung T. Hogberg H. T. Kleensang A. Zhao L. (2013). Review: toxicometabolomics. *J. Appl. Toxicol.* 33 1365–1383. 10.1002/jat.2874 23722930 PMC3808515

[B16] Bouhifd M. Hogberg H. T. Kleensang A. Maertens A. Zhao L. Hartung T. (2014). Mapping the human toxome by systems toxicology. *Basic Clin. Pharmacol. Toxicol.* 115 24–31. 10.1111/bcpt.12198 24443875 PMC4051836

[B17] Braun R. (2014). Systems analysis of high-throughput data. *Adv. Exp. Med. Biol.* 844 153–187. 10.1007/978-1-4939-2095-2_8 25480641 PMC4426208

[B18] Burden N. Sewell F. Andersen M. E. Boobis A. Chipman J. K. Cronin M. T. D. (2015). Adverse outcome pathways can drive non-animal approaches for safety assessment. *J. Appl. Toxicol.* 971–975. 10.1002/jat.3165 25943792 PMC4682468

[B19] Burgoon L. D. Zacharewski T. R. (2008). Automated quantitative dose-response modeling and point of departure determination for large toxicogenomic and high-throughput screening data sets. *Toxicol. Sci.* 104 412–418. 10.1093/toxsci/kfn083 18441342

[B20] Calvani R. Miccheli A. Capuani G. Tomassini Miccheli A. Puccetti C. Delfini M. (2010). Gut microbiome-derived metabolites characterize a peculiar obese urinary metabotype. *Int. J. Obes.* 34 1095–1098. 10.1038/ijo.2010.44 20212498

[B21] Chen R. Snyder M. (2012). Systems biology: personalized medicine for the future? *Curr. Opin. Pharmacol.* 12 623–628. 10.1016/j.coph.2012.07.011 22858243 PMC4076046

[B22] Choucha Snouber L. Bunescu A. Naudot M. Legallais C. Brochot C. Dumas M. E. (2013). Metabolomics-on-a-chip of hepatotoxicity induced by anticancer drug flutamide and Its active metabolite hydroxyflutamide using HepG2/C3a microfluidic biochips. *Toxicol. Sci.* 132 8–20. 10.1093/toxsci/kfs230 22843567

[B23] Clarke C. J. Haselden J. N. (2008). Metabolic profiling as a tool for understanding mechanisms of toxicity. *Toxicol. Pathol.* 36 140–147. 10.1177/0192623307310947 18337232

[B24] Clayton T. A. Lindon J. C. Cloarec O. Antti H. Charuel C. Hanton G. (2006). Pharmaco-metabonomic phenotyping and personalized drug treatment. *Nature* 440 1073–1077. 10.1038/nature04648 16625200

[B25] Clewell H. J. Tan Y. M. Campbell J. L. Andersen M. E. (2008). Quantitative interpretation of human biomonitoring data. *Toxicol. Appl. Pharmacol.* 231 122–133. 10.1016/j.taap.2008.04.021 18589468

[B26] Clewell R. A. Clewell H. J. III (2008). Development and specification of physiologically based pharmacokinetic models for use in risk assessment. *Reg. Toxicol. Pharmacol.* 50 129–143. 10.1016/j.yrtph.2007.10.012 18077066

[B27] Coecke S. Pelkonen O. Leite S. B. Bernauer U. Bessems J. G. Bois F. Y. (2013). Toxicokinetics as a key to the integrated toxicity risk assessment based primarily on non-animal approaches. *Toxicol. In Vitro* 27 1570–1577. 10.1016/j.tiv.2012.06.012 22771339

[B28] Coen M. Holmes E. Lindon J. C. Nicholson J. K. (2008). NMR-based metabolic profiling and metabonomic approaches to problems in molecular toxicology. *Chem. Res. Toxicol.* 21 9–27. 10.1021/tx700335d 18171018

[B29] Collins F. S. (2004). The case for a US prospective cohort study of genes and environment. *Nature* 429 475–477. 10.1038/nature02628 15164074

[B30] Collins F. S. Varmus H. (2015). A new initiative on precision medicine. *N. Engl. J. Med.* 372 793–795. 10.1056/NEJMp1500523 25635347 PMC5101938

[B31] Conolly R. B. Butterworth B. E. (1995). Biologically based dose response model for hepatic toxicity: a mechanistically based replacement for traditional estimates of noncancer risk. *Toxicol. Lett.* 82–83, 901–906. 10.1016/0378-4274(95)03528-1 8597160

[B32] Cote I. Burgoon L. DeWoskin R. (2014). *Next Generation Risk Assessment: Incorporation of Recent Advances in Molecular, Computational, and Systems Biology.* Final Report EPA/600/R-14/004 Washington, DC: U.S. Environmental Protection Agency.

[B33] De Wever B. Goldberg A. Eskes C. Roggen E. Vanparys P. Schröder K. (2015). “Open source”–based engineered human tissue models: a new gold standard for nonanimal testing through openness, transparency, and collaboration, promoted by the ALEXANDRA Association. *Appl. In Vitro Toxicology* 1 5–9. 10.1089/aivt.2014.0011

[B34] Dearry A. (2013). Integrating environmental health data to advance discovery. *Paper Presented at the Emerging Science for Environmental Health Decisions* Washington, DC.

[B35] Debnath J. Muthuswamy S. K. Brugge J. S. (2003). Morphogenesis and oncogenesis of MCF-10A mammary epithelial acini grown in three-dimensional basement membrane cultures. *Methods* 30 256–268. 10.1016/S1046-2023(03)00032-X 12798140

[B36] Dudka I. Kossowska B. Senhadri H. Latajka R. Hajek J. Andrzejak R. (2014). Metabonomic analysis of serum of workers occupationally exposed to arsenic, cadmium and lead for biomarker research: a preliminary study. *Environ. Int.* 68 71–81. 10.1016/j.envint.2014.03.015 24713610

[B37] Dumas M. E. (2012). Metabolome 2.0: quantitative genetics and network biology of metabolic phenotypes. *Mol. BioSyst.* 8 2494–2502. 10.1039/c2mb25167a 22868675

[B38] Edwards S. W. Tan Y. M. Villeneuve D. L. Meek M. E. McQueen C. A. (2016). Adverse outcome pathways-organizing toxicological information to improve decision making. *J. Pharmacol. Exp. Ther.* 356 170–181. 10.1124/jpet.115.228239 26537250

[B39] Eisenstein M. (2015). Big data: the power of petabytes. *Nature* 527 S2–S4. 10.1038/527S2a 26536222

[B40] Ellis J. K. Athersuch T. J. Thomas L. D. Teichert F. Perez-Trujillo M. Svendsen C. (2012). Metabolic profiling detects early effects of environmental and lifestyle exposure to cadmium in a human population. *BMC Med.* 10:61 10.1186/1741-7015-10-61 PMC3391181 22713677

[B41] European Food Safety Authority (2014). Modern methodologies and tools for human hazard assessment of chemicals. *EFSA J.* 12 1–87.

[B42] Fan R. Vermesh O. Srivastava A. Yen B. K. Qin L. Ahmad H. (2008). Integrated barcode chips for rapid, multiplexed analysis of proteins in microliter quantities of blood. *Nat. Biotechnol.* 26 1373–1378. 10.1038/nbt.1507 19029914 PMC2775523

[B43] Feinberg A. P. (2010). Epigenomics reveals a functional genome anatomy and a new approach to common disease. *Nat. Biotechnol.* 28 1049–1052. 10.1038/nbt1010-1049 20944596 PMC2956605

[B44] Feinberg A. P. Irizarry R. A. Fradin D. Aryee M. J. Murakami P. Aspelund T. (2010). Personalized epigenomic signatures that are stable over time and covary with body mass index. *Sci. Transl. Med.* 2:49ra67 10.1126/scitranslmed.3001262 PMC3137242 20844285

[B45] Fentem J. Chamberlain M. Sangster B. (2004). The feasibility of replacing animal testing for assessing consumer safety: a suggested future direction. *Altern. Lab. Anim.* 32 617–623. 15757499 10.1177/026119290403200612

[B46] Fiehn O. (2001). Combining genomics, metabolome analysis, and biochemical modelling to understand metabolic networks. *Comp. Funct. Genomics* 2 155–168. 10.1002/cfg.82 18628911 PMC2447208

[B47] Galas D. J. Hood L. (2009). Systems biology and emerging technologies will catalyze the transition from reactive medicine to predictive, personalized, preventive and participatory (P4) medicine. *IBC* 1:26.

[B48] Gao W. Emaminejad S. Nyein H. Y. Y. Challa S. Chen K. Peck A. (2016). Fully integrated wearable sensor arrays for multiplexed in situ perspiration analysis. *Nature* 529 509–514. 10.1038/nature16521 26819044 PMC4996079

[B49] Gao Y. Lu Y. Huang S. Gao L. Liang X. Wu Y. (2014). Identifying early urinary metabolic changes with long-term environmental exposure to cadmium by mass-spectrometry-based metabolomics. *Environ. Sci. Technol.* 48 6409–6418. 10.1021/es500750w 24834460

[B50] Gasparri R. Santonico M. Valentini C. Sedda G. Borri A. Petrella F. (2016). Volatile signature for the early diagnosis of lung cancer. *J. Breath Res.* 10:016007. 10.1088/1752-7155/10/1/016007 26857451

[B51] Gill S. R. Pop M. Deboy R. T. Eckburg P. B. Turnbaugh P. J. Samuel B. S. (2006). Metagenomic analysis of the human distal gut microbiome. *Science* 312 1355–1359. 10.1126/science.1124234 16741115 PMC3027896

[B52] Griffin J. L. Bollard M. E. (2004). Metabonomics: its potential as a tool in toxicology for safety assessment and data integration. *Curr. Drug Metab.* 5 389–398. 10.2174/1389200043335432 15544433

[B53] Guarner F. Malagelada J. R. (2003). Gut flora in health and disease. *Lancet* 361 512–519. 10.1016/S0140-6736(03)12489-0 12583961

[B54] Guo L. Milburn M. V. Ryals J. A. Lonergan S. C. Mitchell M. W. Wulff J. E. (2015). Plasma metabolomic profiles enhance precision medicine for volunteers of normal health. *Proc. Natl. Acad. Sci. U.S.A.* 112 E4901–E4910. 10.1073/pnas.1508425112 26283345 PMC4568216

[B55] Harrill A. H. Rusyn I. (2008). Systems biology and functional genomics approaches for the identification of cellular responses to drug toxicity. *Expert. Opin. Drug Metab. Toxicol.* 4 1379–1389. 10.1517/17425255.4.11.1379 18950280 PMC2614284

[B56] Heinemann J. Noon B. Mohigmi M. J. Mazurie A. Dickensheets D. L. Bothner B. (2014). Real-time digitization of metabolomics patterns from a living system using mass spectrometry. *J. Am. Soc. Mass Spect.* 25 1755–1762. 10.1007/s13361-014-0922-z PMC4163111 25001378

[B57] Holmes E. Holmes and Nicholson J. K. (2007). Human metabolic phenotyping and metabolome wide association studies. *Ernst Schering Found. Symp. Proc.* 227–249. 18811060 10.1007/2789_2008_096

[B58] Holmes E. Wilson I. D. Nicholson J. K. (2008). Metabolic phenotyping in health and disease. *Cell* 134 714–717. 10.1016/j.cell.2008.08.026 18775301

[B59] Hood L. Balling R. Auffray C. (2012). Revolutionizing medicine in the 21st century through systems approaches. *Biotechnol. J.* 7 992–1001. 10.1002/biot.201100306 22815171 PMC3962497

[B60] Hood L. Flores M. A. Brogaard K. R. Price N. D. (2013). “Systems medicine and the emergence of proactive p4 medicine: predictive, preventive, personalized and participatory a2 - Dekker,” in *Handbook of Systems Biology* eds Marian Walhout >A. J. Vidal M. Dekker J. (San Diego, CA: Academic Press), 445–467.

[B61] Hood L. Friend S. H. (2011). Predictive, personalized, preventive, participatory (P4) cancer medicine. *Nat. Rev. Clin. Oncol.* 8 184–187. 10.1038/nrclinonc.2010.227 21364692

[B62] Hood L. Lovejoy J. C. Price N. D. (2015). Integrating big data and actionable health coaching to optimize wellness. *BMC Med.* 13:4 10.1186/s12916-014-0238-7 PMC4288554 25575752

[B63] Huang R. Xia M. Sakamuru S. Zhao J. Shahane S. A. Attene-Ramos M. (2016). Modelling the Tox21 10[thinsp]K chemical profiles for in vivo toxicity prediction and mechanism characterization. *Nat. Commun.* 7:10425 10.1038/ncomms10425 PMC4777217 26811972

[B64] Huh D. Kim H. J. Fraser J. P. Shea D. E. Khan M. Bahinski A. (2013). Microfabrication of human organs-on-chips. *Nat. Protoc.* 8 2135–2157. 10.1038/nprot.2013.137 24113786

[B65] Huh D. Matthews B. D. Mammoto A. Montoya-Zavala M. Hsin H. Y. Ingber D. E. (2010). Reconstituting organ-level lung functions on a chip. *Science* 328 1662–1668. 10.1126/science.1188302 20576885 PMC8335790

[B66] Huh D. Torisawa Y. S. Hamilton G. A. Kim H. J. Ingber D. E. (2012). Microengineered physiological biomimicry: organs-on-chips. *Lab. Chip* 12 2156–2164. 10.1039/c2lc40089h 22555377

[B67] IGHRC (1999). *Physiologically-based Pharmacokinetic Modelling: A Potential Tool for Use in risk Assessment*. Available at: http://www.iehconsulting.co.uk/IEH_Consulting/IEHCPubs/IGHRC/cr4.pdf

[B68] Jack J. Wambaugh J. Shah I. (2013). Systems toxicology from genes to organs. *Methods Mol. Biol.* 930 375–397. 10.1007/978-1-62703-059-5_17 23086851

[B69] Jansen K. A. Donato D. M. Balcioglu H. E. Schmidt T. Danen E. H. Koenderink G. H. (2015). A guide to mechanobiology: where biology and physics meet. *Biochim. Biophys. Acta* 1853(Pt B) 3043–3052. 10.1016/j.bbamcr.2015.05.007 25997671

[B70] Johanson G. Jonsson F. Bois F. (1999). Development of new technique for risk assessment using physiologically based toxicokinetic models. Am. J. Ind. Med., 101–103. 10.1002/(sici)1097-0274(199909)36:1+<101::aid-ajim36>3.0.co;2-i 10519801

[B71] Jones D. P. Park Y. Ziegler T. R. (2012). Nutritional metabolomics: progress in addressing complexity in diet and health. *Annu. Rev. Nutr.* 32 183–202. 10.1146/annurev-nutr-072610-145159 22540256 PMC4031100

[B72] Kamburov A. Stelzl U. Lehrach H. Herwig R. (2013). The ConsensusPathDB interaction database: 2013 update. *Nucleic Acids Res.* 41 D793–D800. 10.1093/nar/gks1055 23143270 PMC3531102

[B73] Kankainen M. Gopalacharyulu P. Holm L. Oresic M. (2011). MPEA–metabolite pathway enrichment analysis. *Bioinformatics* 27 1878–1879. 10.1093/bioinformatics/btr278 21551139

[B74] Keun H. C. Ebbels T. M. Antti H. Bollard M. E. Beckonert O. Schlotterbeck G. (2002). Analytical reproducibility in (1)H NMR-based metabonomic urinalysis. *Chem. Res. Toxicol.* 15 1380–1386. 10.1021/tx0255774 12437328

[B75] Kim S. Kim W. Wei C. H. Lu Z. Wilbur W. J. (2012). Prioritizing PubMed articles for the comparative toxicogenomic database utilizing semantic information. *Database (Oxford)* 2012:bas042 10.1093/database/bas042 PMC3500521 23160415

[B76] Kinross J. von Roon A. C. Penney N. Holmes E. Silk D. Nicholson J. K. (2009). The gut microbiota as a target for improved surgical outcome and improved patient care. *Curr. Pharm. Des.* 15 1537–1545. 10.2174/138161209788168119 19442171

[B77] Kinross J. M. von Roon A. C. Holmes E. Darzi A. Nicholson J. K. (2008). The human gut microbiome: implications for future health care. *Curr. Gastroenterol. Rep.* 10 396–403. 10.1007/s11894-008-0075-y 18627653

[B78] Konvalina G. Haick H. (2013). Sensors for Breath Testing: From Nanomaterials to Comprehensive Disease Detection. Acc Chem Res. 10.1021/ar400070m 23926883

[B79] Kosmides A. K. Kamisoglu K. Calvano S. E. Corbett S. A. Androulakis I. P. (2013). Metabolomic fingerprinting: challenges and opportunities. *Crit. Rev. Biomed. Eng.* 41 205–221. 10.1615/CritRevBiomedEng.2013007736 24579644 PMC4096240

[B80] Kotera M. Hirakawa M. Tokimatsu T. Goto S. Kanehisa M. (2012). The KEGG databases and tools facilitating omics analysis: latest developments involving human diseases and pharmaceuticals. *Methods Mol. Biol.* 802 19–39. 10.1007/978-1-61779-400-1_2 22130871

[B81] Kraly J. R. Holcomb R. E. Guan Q. Henry C. S. (2009). Review: microfluidic applications in metabolomics and metabolic profiling. *Anal. Chim. Acta* 653 23–35. 10.1016/j.aca.2009.08.037 19800473 PMC2791705

[B82] Krilaviciute A. Heiss J. A. Leja M. Kupcinskas J. Haick H. Brenner H. (2015). Detection of cancer through exhaled breath: a systematic review. *Oncotarget* 6 38643–38657. 10.18632/oncotarget.5938 26440312 PMC4770726

[B83] Kuo C. H. Wang K. C. Tian T. F. Tsai M. H. Chiung Y. M. Hsiech C. M. (2012). Metabolomic characterization of laborers exposed to welding fumes. *Chem. Res. Toxicol.* 25 676–686. 10.1021/tx200465e 22292500

[B84] Leung E. L. Cao Z. W. Jiang Z. H. Zhou H. Liu L. (2013). Network-based drug discovery by integrating systems biology and computational technologies. *Brief. Bioinform.* 14 491–505. 10.1093/bib/bbs043 22877768 PMC3713711

[B85] Li M. Wang B. Zhang M. Rantalainen M. Wang S. Zhou H. (2008). Symbiotic gut microbes modulate human metabolic phenotypes. *Proc. Natl. Acad. Sci. U.S.A.* 105 2117–2122. 10.1073/pnas.0712038105 18252821 PMC2538887

[B86] Lin C. Q. Bissell M. J. (1993). Multi-faceted regulation of cell differentiation by extracellular matrix. *FASEB J.* 7 737–743. 8330681 10.1096/fasebj.7.9.8330681

[B87] Lipscomb J. C. Poet T. S. (2008). In vitro measurements of metabolism for application in pharmacokinetic modeling. *Pharmacol. Ther.* 118 82–103. 18374419 10.1016/j.pharmthera.2008.01.006

[B88] Loizou G. D. Hogg A. (2011). MEGen: A Physiologically Based Pharmacokinetic Model Generator. *Frontiers in Pharmacology: Predictive Toxicity* 2 56 1–14 10.3389/fphar.2011.00056 PMC3212724 22084631

[B89] Loscalzo J. Barabasi A. L. (2011). Systems biology and the future of medicine. *Wiley Interdiscip Rev. Syst. Biol. Med.* 3 619–627. 10.1002/wsbm.144 21928407 PMC3188693

[B90] Loscalzo J. Kohane I. Barabasi A. L. (2007). Human disease classification in the postgenomic era: a complex systems approach to human pathobiology. *Mol. Syst. Biol.* 3:124 10.1038/msb4100163 PMC1948102 17625512

[B91] Louisse J. Verwei M. Woutersen R. A. Blaauboer B. J. Rietjens I. M. (2012). Toward in vitro biomarkers for developmental toxicity and their extrapolation to the in vivo situation. *Expert. Opin. Drug Metab. Toxicol.* 8 11–27. 10.1517/17425255.2012.639762 22114915

[B92] Lyons M. A. Yang R. S. Mayeno A. N. Reisfeld B. (2008). Computational toxicology of chloroform: reverse dosimetry using bayesian inference, markov chain monte carlo simulation, and human biomonitoring data. *Environ. Health Perspect.* 116 1040–1046. 10.1289/ehp.11079 18709138 PMC2516557

[B93] MacFie J. O’Boyle C. Mitchell C. J. Buckley P. M. Johnstone D. Sudworth P. (1999). Gut origin of sepsis: a prospective study investigating associations between bacterial translocation, gastric microflora, and septic morbidity. *Gut* 45 223–228. 10.1136/gut.45.2.223 10403734 PMC1727620

[B94] Maher A. D. Zirah S. F. Holmes E. Nicholson J. K. (2007). Experimental and analytical variation in human urine in 1H NMR spectroscopy-based metabolic phenotyping studies. *Anal. Chem.* 79 5204–5211. 10.1021/ac070212f 17555297

[B95] Mak H. C. (2015). Trends in precision medicine: an interview with UCSF’s Atul Butte. *Cell Syst.* 1 254–255. 10.1016/j.cels.2015.10.007 27136054

[B96] Mastrangelo A. Armitage E. G. Garcia A. Barbas C. (2014). Metabolomics as a tool for drug discovery and personalised medicine. A review. *Curr. Top. Med. Chem.* 14 2627–2636. 10.2174/1568026614666141215124956 25515755

[B97] McNally K. Cotton R. Cocker J. Jones K. Bartels M. Rick D. (2012). Reconstruction of exposure to *m*-Xylene from human biomonitoring data using PBPK modelling, Bayesian inference, and Markov Chain Monte Carlo simulation. *J. Toxicol.* 2012:760281 10.1155/2012/760281 PMC3376947 22719759

[B98] McNally K. Cotton R. Hogg A. Loizou G. (2014). PopGen: a virtual human population generator. *Toxicology* 315 70–85. 10.1016/j.tox.2013.07.009 23876857

[B99] McNally K. Cotton R. Loizou G. (2011). A workflow for global sensitivity analysis of PBPK models. *Front. Pharmacol.* 2:31 10.3389/fphar.2011.00031 PMC3128931 21772819

[B100] Monte A. A. Brocker C. Nebert D. W. Gonzalez F. J. Thompson D. C. Vasiliou V. (2014). Improved drug therapy: triangulating phenomics with genomics and metabolomics. *Hum. Genomics* 8:16 10.1186/s40246-014-0016-9 PMC4445687 25181945

[B101] Moreno E. L. Hachi S. Hemmer K. Trietsch S. J. Baumuratov A. S. Hankemeier T. (2015). Differentiation of neuroepithelial stem cells into functional dopaminergic neurons in 3D microfluidic cell culture. *Lab. Chip* 15 2419–2428. 10.1039/c5lc00180c 25902196

[B102] Nicholson J. K. (2006). Global systems biology, personalized medicine and molecular epidemiology. *Mol. Syst. Biol.* 2:52 10.1038/msb4100095 PMC1682018 17016518

[B103] Nicholson J. K. Holmes E. (2006). Global systems biology and personalized healthcare solutions. *Discov. Med.* 6 63–70. 17234128

[B104] Nicholson J. K. Holmes E. Elliott P. (2008). The metabolome-wide association study: a new look at human disease risk factors. *J. Proteome Res.* 7 3637–3638. 10.1021/pr8005099 18707153

[B105] Nicholson J. K. Holmes E. Kinross J. Burcelin R. Gibson G. Jia W. (2012). Host-gut microbiota metabolic interactions. *Science* 336 1262–1267. 10.1126/science.1223813 22674330

[B106] Nicholson J. K. Holmes E. Lindon J. C. Wilson I. D. (2004). The challenges of modeling mammalian biocomplexity. *Nat. Biotechnol.* 22 1268–1274. 10.1038/nbt1015 15470467

[B107] Nicholson J. K. Holmes E. Wilson I. D. (2005). Gut microorganisms, mammalian metabolism and personalized health care. *Nat. Rev. Microbiol.* 3 431–438. 10.1038/nrmicro1152 15821725

[B108] Nicholson J. K. Lindon J. C. (2008). Systems biology: metabonomics. *Nature* 455 1054–1056. 10.1038/4551054a 18948945

[B109] NRC (2007). *Toxicity Testing in the Twenty-First Century: A Vision and a Strategy*. Washington, DC: National Research Council 146.

[B110] Pamies D. Hartung T. Hogberg H. T. (2014). Biological and medical applications of a brain-on-a-chip. *Exp. Biol. Med.* 239 1096–1107. 10.1177/1535370214537738 PMC4779552 24912505

[B111] Piersma A. H. Ezendam J. Luijten M. Muller J. J. Rorije E. van der Ven L. T. (2014). A critical appraisal of the process of regulatory implementation of novel in vivo and in vitro methods for chemical hazard and risk assessment. *Crit. Rev. Toxicol.* 44 876–894. 10.3109/10408444.2014.940445 25058877

[B112] Posma J. M. Robinette S. L. Holmes E. Nicholson J. K. (2014). MetaboNetworks, an interactive Matlab-based toolbox for creating, customizing and exploring sub-networks from KEGG. *Bioinformatics* 30 893–895. 10.1093/bioinformatics/btt612 24177720 PMC3957072

[B113] Prot J. M. Aninat C. Griscom L. Razan F. Brochot C. Guillouzo C. G. (2011). Improvement of HepG2/C3a cell functions in a microfluidic biochip. *Biotechnol. Bioeng.* 108 1704–1715. 10.1002/bit.23104 21337338

[B114] Prot J. M. Bunescu A. Elena-Herrmann B. Aninat C. Snouber L. C. Griscom L. (2012). Predictive toxicology using systemic biology and liver microfluidic “on chip” approaches: application to acetaminophen injury. *Toxicol. Appl. Pharmacol.* 259 270–280. 10.1016/j.taap.2011.12.017 22230336

[B115] Prot J. M. Leclerc E. (2012). The current status of alternatives to animal testing and predictive toxicology methods using liver microfluidic biochips. *Ann. Biomed. Eng.* 40 1228–1243. 10.1007/s10439-011-0480-5 22160577

[B116] Raamsdonk L. M. Teusink B. Broadhurst D. Zhang N. Hayes A. Walsh M. C. (2001). A functional genomics strategy that uses metabolome data to reveal the phenotype of silent mutations. *Nat. Biotechnol.* 19 45–50. 11135551 10.1038/83496

[B117] Rabinowitz J. D. Purdy J. G. Vastag L. Shenk T. Koyuncu E. (2011). Metabolomics in drug target discovery. *Cold. Spring Harb. Symp. Quant. Biol.* 76 235–246. 10.1101/sqb.2011.76.010694 22114327 PMC4084595

[B118] Rattray N. J. Hamrang Z. Trivedi D. K. Goodacre R. Fowler S. J. (2014). Taking your breath away: metabolomics breathes life in to personalized medicine. *Trends Biotechnol.* 32 538–548. 10.1016/j.tibtech.2014.08.003 25179940

[B119] Rehm H. L. Berg J. S. Brooks L. D. Bustamante C. D. Evans J. P. Landrum M. J. (2015). ClinGen–the clinical genome resource. *N. Engl. J. Med.* 372 2235–2242. 10.1056/NEJMsr1406261 26014595 PMC4474187

[B120] Sakurai N. Ara T. Ogata Y. Sano R. Ohno T. Sugiyama K. (2011). KaPPA-View4: a metabolic pathway database for representation and analysis of correlation networks of gene co-expression and metabolite co-accumulation and omics data. *Nucleic Acids Res.* 39 D677–D684. 10.1093/nar/gkq989 21097783 PMC3013809

[B121] Sand S. Ringblom J. Hakansson H. Oberg M. (2012). The point of transition on the dose-effect curve as a reference point in the evaluation of in vitro toxicity data. *J. Appl. Toxicol.* 32 843–849. 10.1002/jat.2757 22733407

[B122] Sand S. von Rosen D. Victorin K. Filipsson A. F. (2006). Identification of a critical dose level for risk assessment: developments in benchmark dose analysis of continuous endpoints. *Toxicol. Sci.* 90 241–251. 10.1093/toxsci/kfj057 16322076

[B123] Schoenenberger C. A. Zuk A. Zinkl G. M. Kendall D. Matlin K. S. (1994). Integrin expression and localization in normal MDCK cells and transformed MDCK cells lacking apical polarity. *J. Cell Sci.* 107(Pt 2), 527–541. 7515897 10.1242/jcs.107.2.527

[B124] Schumacher A. Rujan T. Hoefkens J. (2014). A collaborative approach to develop a multi-omics data analytics platform for translational research. *Appl. Transl. Genom.* 3 105–108. 10.1016/j.atg.2014.09.010 27294023 PMC4888831

[B125] Serkova N. J. Standiford T. J. Stringer K. A. (2011). The emerging field of quantitative blood metabolomics for biomarker discovery in critical illnesses. *Am. J. Respir. Crit. Care Med.* 184 647–655. 10.1164/rccm.201103-0474CI 21680948 PMC3208597

[B126] Shen H. Xu W. Zhang J. Chen M. Martin F. L. Xia Y. (2013). Urinary metabolic biomarkers link oxidative stress indicators associated with general arsenic exposure to male infertility in a han chinese population. *Environ. Sci. Technol.* 47 8843–8851. 10.1021/es402025n 23841501

[B127] Shimizu K. Ogura H. Goto M. Asahara T. Nomoto K. Morotomi M. (2006). Altered gut flora and environment in patients with severe SIRS. *J. Trauma* 60 126–133. 10.1097/01.ta.0000197374.99755.fe 16456446

[B128] Shintu L. Baudoin R. Navratil V. Prot J. M. Pontoizeau C. Defernez M. (2012). Metabolomics-on-a-chip and predictive systems toxicology in microfluidic bioartificial organs. *Anal. Chem.* 84 1840–1848. 10.1021/ac2011075 22242722

[B129] Siva N. (2015). UK gears up to decode 100,000 genomes from NHS patients. *Lancet* 385 103–104. 25540888 10.1016/S0140-6736(14)62453-3

[B130] Smarr L. (2012). Quantifying your body: a how-to guide from a systems biology perspective. *Biotechnol. J.* 7 980–991. 10.1002/biot.201100495 22887886

[B131] Smith A. S. Long C. J. Berry B. J. McAleer C. Stancescu M. Molnar P. (2013). Microphysiological systems and low-cost microfluidic platform with analytics. *Stem Cell Res. Ther.* 4(Suppl. 1) S9 10.1186/scrt370 PMC4029761 24565109

[B132] Sobradillo P. Pozo F. Agusti A. (2011). P4 medicine: the future around the corner. *Arch. Bronconeumol.* 47 35–40. 10.1016/j.arbres.2010.09.009 21190770

[B133] Sonner Z. Wilder E. Heikenfeld J. Kasting G. Beyette F. Swaile D. (2015). The microfluidics of the eccrine sweat gland, including biomarker partitioning, transport, and biosensing implications. *Biomicrofluidics* 9:031301 10.1063/1.4921039 PMC4433483 26045728

[B134] Stanberry L. Mias G. I. Haynes W. Higdon R. Snyder M. Kolker E. (2013). Integrative analysis of longitudinal metabolomics data from a personal multi-omics profile. *Metabolites* 3 741–760. 10.3390/metabo3030741 24958148 PMC3901289

[B135] Stobbe M. D. Jansen G. A. Moerland P. D. van Kampen A. H. C. (2014). Knowledge representation in metabolic pathway databases. *Brief. Bioinform.* 15 455–470. 10.1093/bib/bbs060 23202525

[B136] Sturla S. J. Boobis A. R. FitzGerald R. E. Hoeng J. Kavlock R. J. Schirmer K. (2014). Systems toxicology: from basic research to risk assessment. *Chem. Res. Toxicol.* 27 314–329. 10.1021/tx400410s 24446777 PMC3964730

[B137] Sulek K. Han T. L. Villas-Boas S. G. Wishart D. S. Soh S. E. Kwek K. (2014). Hair metabolomics: identification of fetal compromise provides proof of concept for biomarker discovery. *Theranostics* 4 953–959. 10.7150/thno.9265 25057319 PMC4107295

[B138] Tan Y. M. Liao K. H. Clewell H. J. III. (2006). Reverse dosimetry: interpreting trihalomethanes biomonitoring data using physiologically based pharmacokinetic modeling. *J. Exp. Sci. Environ. Epidemiol.* 17 591–603. 10.1038/sj.jes.7500540 17108893

[B139] Thomas R. S. Philbert M. A. Auerbach S. S. Wetmore B. A. Devito M. J. Cote I. (2013). Incorporating new technologies into toxicity testing and risk assessment: moving from 21st century vision to a data-driven framework. *Toxicol. Sci.* 136 4–18. 10.1093/toxsci/kft178 23958734 PMC3829570

[B140] Topol E. J. (2010). Transforming medicine via digital innovation. *Sci. Transl. Med.* 2:16cm4 10.1126/scitranslmed.3000484 PMC3756088 20371472

[B141] Topol E. J. (2012). *The Creative Destruction of Medicine: How the Digital Revolution Will Create Better Health Care*. New York, NY: Basic Books.

[B142] Topol E. J. (2014). Individualized medicine from prewomb to tomb. *Cell* 157 241–253. 10.1016/j.cell.2014.02.012 24679539 PMC3995127

[B143] Topol E. J. Steinhubl S. R. Torkamani A. (2015). Digital medical tools and sensors. *JAMA* 313 353–354. 10.1001/jama.2014.17125 25626031 PMC4751024

[B144] Trietsch S. J. Israels G. D. Joore J. Hankemeier T. Vulto P. (2013). Microfluidic titer plate for stratified 3D cell culture. *Lab. Chip* 13 3548–3554. 10.1039/c3lc50210d 23887749

[B145] Tseng P. Weaver W. M. Masaeli M. Owsley K. Di Carlo D. (2014). Research highlights: microfluidics meets big data. *Lab. Chip* 14 828–832. 10.1039/c4lc90001d 24473594

[B146] Turnbaugh P. J. Ley R. E. Mahowald M. A. Magrini V. Mardis E. R. Gordon J. I. (2006). An obesity-associated gut microbiome with increased capacity for energy harvest. *Nature* 444 1027–1031. 10.1038/nature05414 17183312

[B147] van Duinen V. Trietsch S. J. Joore J. Vulto P. Hankemeier T. (2015). Microfluidic 3D cell culture: from tools to tissue models. *Curr. Opin. Biotechnol.* 35 118–126. 10.1016/j.copbio.2015.05.002 26094109

[B148] Veenstra T. D. (2012). Metabolomics: the final frontier? *Genome Med* 4:40 10.1186/gm339 PMC3446268 22546050

[B149] Vinken M. (2013). The adverse outcome pathway concept: a pragmatic tool in toxicology. *Toxicology* 312 158–165. 10.1016/j.tox.2013.08.011 23978457

[B150] Wachter A. Beissbarth T. (2015). pwOmics: an R package for pathway-based integration of time-series omics data using public database knowledge. *Bioinformatics* 31 3072–3074. 10.1093/bioinformatics/btv323 26002883

[B151] Wambaugh J. F. Setzer R. W. Reif D. M. Gangwal S. Mitchell-Blackwood J. Arnot J. A. (2013). High-throughput models for exposure-based chemical prioritization in the ExpoCast project. *Environ. Sci. Technol.* 47 8479–8488. 10.1021/es400482g 23758710

[B152] Wambaugh J. F. Wang A. Dionisio K. L. Frame A. Egeghy P. Judson R. (2014). High Throughput Heuristics for Prioritizing Human Exposure to Environmental Chemicals. *Environ. Sci. Technol.* 48 12760–12767. 10.1021/es503583j 25343693

[B153] Wambaugh J. F. Wetmore B. A. Pearce R. Strope C. Goldsmith R. Sluka J. P. (2015). Toxicokinetic triage for environmental chemicals. *Toxicol. Sci.* 147 55–67. 10.1093/toxsci/kfv118 26085347 PMC4560038

[B154] Wang Z. Zheng Y. Zhao B. Zhang Y. Liu Z. Xu J. (2015). Human metabolic responses to chronic environmental polycyclic aromatic hydrocarbon exposure by a metabolomic approach. *J. Proteome Res.* 14 2583–2593. 10.1021/acs.jproteome.5b00134 25990285

[B155] Weaver V. M. Petersen O. W. Wang F. Larabell C. A. Briand P. Damsky C. (1997). Reversion of the malignant phenotype of human breast cells in three-dimensional culture and in vivo by integrin blocking antibodies. *J. Cell Biol.* 137 231–245. 10.1083/jcb.137.1.231 9105051 PMC2139858

[B156] Wetmore B. A. Wambaugh J. F. Allen B. Ferguson S. S. Sochaski M. A. Setzer R. W. (2015). Incorporating high-throughput exposure predictions with dosimetry-adjusted *in vitro* bioactivity to inform chemical toxicity testing. *Toxicol. Sci.* 148 121–136. 10.1093/toxsci/kfv171 26251325 PMC4620046

[B157] Wikoff W. R. Anfora A. T. Liu J. Schultz P. G. Lesley S. A. Peters E. C. (2009). Metabolomics analysis reveals large effects of gut microflora on mammalian blood metabolites. *Proc. Natl. Acad. Sci. U.S.A.* 106 3698–3703. 10.1073/pnas.0812874106 19234110 PMC2656143

[B158] Wikoff W. R. Frye R. F. Zhu H. Gong Y. Boyle S. Churchill E. (2013). Pharmacometabolomics reveals racial differences in response to atenolol treatment. *PLoS ONE* 8:e57639 10.1371/journal.pone.0057639 PMC3594230 23536766

[B159] Williams A. J. Harland L. Groth P. Pettifer S. Chichester C. Willighagen E. L. (2012). Open PHACTS: semantic interoperability for drug discovery. *Drug Discov. Today* 17 1188–1198. 10.1016/j.drudis.2012.05.016 22683805

[B160] Wishart D. S. (2007). Current progress in computational metabolomics. *Brief. Bioinform.* 8 279–293. 10.1093/bib/bbm030 17626065

[B161] Wishart D. S. (2012). Chapter 3: Small molecules and disease. *PLoS Comput. Biol.* 8:e1002805 10.1371/journal.pcbi.1002805 PMC3531289 23300405

[B162] Wishart D. S. Jewison T. Guo A. C. Wilson M. Knox C. Liu Y. (2013). HMDB 3.0–The Human Metabolome Database in 2013. *Nucleic Acids Res.* 41 D801–D807. 10.1093/nar/gks1065 23161693 PMC3531200

[B163] Wishart D. S. Knox C. Guo A. C. Eisner R. Young N. Gautam B. (2009). HMDB: a knowledgebase for the human metabolome. *Nucleic Acids Res.* 37 D603–D610. 10.1093/nar/gkn810 18953024 PMC2686599

[B164] Wishart D. S. Mandal R. Stanislaus A. Ramirez-Gaona M. (2016). Cancer metabolomics and the human metabolome database. *Metabolites* 6 10.3390/metabo6010010 PMC4812339 26950159

[B165] Witze A. (2014). Joint effort nabs next wave of US supercomputers. *Nature* 515 324–324. 10.1038/nature.2014.16347 25409808

[B166] Xia J. Wishart D. S. (2010a). MetPA: a web-based metabolomics tool for pathway analysis and visualization. *Bioinformatics* 26 2342–2344. 10.1093/bioinformatics/btq418 20628077

[B167] Xia J. Wishart D. S. (2010b). MSEA: a web-based tool to identify biologically meaningful patterns in quantitative metabolomic data. *Nucleic Acids Res.* 38 W71–W77. 10.1093/nar/gkq329 20457745 PMC2896187

[B168] Zhang A. Sun H. Han Y. Yan G. Wang X. (2013a). Urinary metabolic biomarker and pathway study of hepatitis B virus infected patients based on UPLC-MS system. *PLoS ONE* 8:e64381 10.1371/journal.pone.0064381 PMC3655955 23696887

[B169] Zhang A. Sun H. Xu H. Qiu S. Wang X. (2013b). Cell metabolomics. *Omics* 17 495–501. 10.1089/omi.2012.0090 23988149 PMC3783970

[B170] Zhang Q. Bhattacharya S. Andersen M. E. Conolly R. B. (2010). Computational systems biology and dose-response modeling in relation to new directions in toxicity testing. *J. Toxicol. Environ. Health B. Crit. Rev.* 13 253–276. 10.1080/10937404.2010.483943 20574901

[B171] Zhu Y. Davis S. Stephens R. Meltzer P. S. Chen Y. (2008). GEOmetadb: powerful alternative search engine for the Gene Expression Omnibus. *Bioinformatics* 24 2798–2800. 10.1093/bioinformatics/btn520 18842599 PMC2639278

[B172] Zhukova A. Sherman D. J. (2014). Knowledge-based generalization of metabolic models. *J. Comput. Biol.* 21 534–547. 10.1089/cmb.2013.0143 24766276

